# Genetic Interactions in Various Environmental Conditions in *Caenorhabditis elegans*

**DOI:** 10.3390/genes14112080

**Published:** 2023-11-15

**Authors:** Katarzyna Toch, Mateusz Buczek, Marta K. Labocha

**Affiliations:** Institute of Environmental Sciences, Faculty of Biology, Jagiellonian University, Ul. Gronostajowa 7, 30-387 Krakow, Poland; katarzyna.toch@gmail.com (K.T.); mateusz.buczek@uj.edu.pl (M.B.)

**Keywords:** genetic interactions, gene networks, environmental stress, epistasis, multicellular

## Abstract

Although it is well known that epistasis plays an important role in many evolutionary processes (e.g., speciation, evolution of sex), our knowledge on the frequency and prevalent sign of epistatic interactions is mainly limited to unicellular organisms or cell cultures of multicellular organisms. This is even more pronounced in regard to how the environment can influence genetic interactions. To broaden our knowledge in that respect we studied gene–gene interactions in a whole multicellular organism, *Caenorhabditis elegans*. We screened over one thousand gene interactions, each one in standard laboratory conditions, and under three different stressors: heat shock, oxidative stress, and genotoxic stress. Depending on the condition, between 7% and 22% of gene pairs showed significant genetic interactions and an overall sign of epistasis changed depending on the condition. Sign epistasis was quite common, but reciprocal sign epistasis was extremally rare. One interaction was common to all conditions, whereas 78% of interactions were specific to only one environment. Although epistatic interactions are quite common, their impact on evolutionary processes will strongly depend on environmental factors.

## 1. Introduction

The ultimate goal of genetics is deciphering how a gene translates into a trait. But not a single gene is a lonely island, and epistasis, the dependence of one locus on other loci in a genetic background, is a long-known phenomenon. Epistatic interactions, though long neglected, are well acknowledged to play an important role in evolutionary processes, such as speciation [1], evolution of sex and recombination [2], selection [3,4], and shaping evolutionary landscapes [5]. Moreover, the general interpretation of an interplay between genotype and phenotype is further complicated by gene–environment interactions. Living organisms are constantly assaulted by endogenous and exogenous stressors in nature and not only can the single-gene effect change under different conditions [6], but it is known that epistatic interactions can change under environmental perturbations as well [7,8,9,10].

Most importantly it is argued that epistasis can have strong effects on the response to directional selection [3,4,11,12,13,14]. But for epistasis to play an important role in natural selection, it is essential that epistasis will be directional—that is, it will be either positive or negative. In case of a negative epistatic interaction (when a combination of deleterious mutations shows a phenotype value that is more severe than expected based on the assumption of additive interactions between genes), the total harm of multiple mutations is enhanced so the effectiveness of natural selection to remove deleterious mutations will increase. The effect will be opposite in the case of a positive epistatic interaction (when a combination of deleterious mutations shows a phenotype value that is milder than expected). In that case, the total harm of multiple deleterious mutations will be reduced and the efficacy of natural selection to eliminate deleterious mutations will decline. When epistasis does not have any systematic direction, it will not influence the rate of evolution [3,14,15].

So, the first important question is this: what is the prevalent sign of epistasis? The second one is this: will the average sign of epistasis change with a changing environment? If so, then the influence of epistasis on natural selection will change as well. Although large-scale experimental platforms for studying epistasis have been developed lately, most of them were applied in studies of gene interactions in single, standard laboratory conditions, and they were limited to unicellular organisms, like bacteria or yeast, or single cells of a multicellular organism [16,17,18,19,20]. In most of those studies, negative epistasis was prevalent [7,10,18,21], although not exclusively [20]. Even less is known about how epistasis will change under environmental perturbations. To date, some studies observed increasing positive epistasis under harsh conditions [7,8,21,22,23], whereas others found opposite trends [10,24]. The contrasting results of those studies are not truly surprising as they encompass different organisms, various and in most cases small sets of interacting genes, and different stressors. Predictions based on Flux Balance Analysis in yeast [15] are that epistasis tends to become more positive in harsh conditions. This prediction was partially confirmed by a study in yeast [9]. The generality of this rule needs to be validated for other organisms, especially multicellular ones, as the analysis performed by Barker and colleagues [15] was based on the data for yeast. So far, dynamic epistasis studies in multicellular organisms have been limited to the network comprising only a few genes [25], and they did not allow the authors to draw conclusions on the differences/similarities of dynamics of epistasis between uni- and multicellular organisms.

To answer the above questions regarding multicellular organisms, we conducted a large-scale epistasis study using a model organism, nematode *C. elegans*.

## 2. Materials and Methods

### 2.1. Query Genes

We searched the WormBase database (225) for genes associated with response to oxidative stress, response to heat, response to cold, and DNA repair, based on results from previous studies listed in WormBase (225). This list comprised over 300 genes. From those, we excluded genes for which no single, homozygous, viable mutants were available. From that final list of over 150 genes, we have preferentially chosen genes for which outcrossed mutants were available. Those genes were associated with a total of over 90 different GO terms.

### 2.2. C. elegans Strains

Outcrossed worm strains were acquired from individual laboratories or *Caenorhabditis* Genetics Center and frozen at −80 °C after delivery (Supplementary Table S1). Animals were maintained at 20 °C on standard nematode growth media (NGM) agar plates seeded with OP50 strain of *Escherichia coli* as a food source [26]. Our final list of worm strains used comprised 26 single gene mutant strains plus wild-type N2 strain (Supplementary Table S1).

### 2.3. RNA Interference and Bacteria Preparation

Single-mutant strains of *C. elegans* were fed with RNAi bacteria producing dsRNA to obtain double mutants [27]. We used *E. coli* RNAi clones from *C. elegans* RNAi Ahringer library (Source BioScience, Supplementary Table S2). Our final list of RNAi strains used comprised 47 RNAi strains (Supplementary Table S2). Additionally, we used HT115 (DE3) as control bacteria with an empty vector.

RNAi bacteria clones were inoculated in 5 mL of LB medium with ampicillin (50 µg/mL of final concentration) and incubated for 15 h at 37 °C with shaking. Next, then they were induced with isopropyl-β-D-thiogalactopyranoside (IPTG; 953 µg/mL of final concentration) to produce RNA and incubated for another hour in the same conditions. After growth bacteria were spun down for 40 min at 3700 rpm and the supernatant was discarded. Bacteria pellets were re-suspended in 900 µL of S Medium with ampicillin and IPTG (final concentration of 100 µg/mL and 953 µg/mL, respectively). Optical density OD (at λ = 600 nm) of re-suspended bacteria was measured (Sunrise, Tecan, Grödig, Austria) and bacteria were diluted to reach a final OD of 0.85. Resuspended bacteria were dispensed into 96-well experimental plates (50 µL of bacteria solution in each well).

### 2.4. Worm Preparation

Gravid adult worms were bleached (with standard NaOH and sodium hypochlorite method; Stiernagle 2006) and eggs were dispensed onto unseeded NGM plates to hatch overnight. The next day starved L1 worms were washed with S Medium with the addition of ampicillin and IPTG (final concentration of 100 µg/mL and 953 µg/mL, respectively) and dispensed (20 µL) onto the experimental plates at a final concentration of approximately 20 worms per well (with final volume of 70 µL of liquid per well).

### 2.5. Fitness Assay

On day 0, in the afternoon, animals were dispensed onto the experimental plates. On day 2, at L4 larval stage, animals were treated with stressors. To each well, 10 µL of solution was added. For MMS treatment, we added 0.016% methyl-methanesulfonate solution, and for H_2_O_2_ treatment, we added 4% hydrogen peroxidase solution. S Medium with the addition of ampicillin and IPTG (final concentration of 100 µg/mL and 953 µg/mL, respectively) was used as a solvent. To control plates, only 10 µL of solvent was added. The first OD reading was taken on day 1 (18 h after L1 larvae were dispensed onto the experimental plates), and the last one was taken five days later, on day 6. Immediately before OD reading, plates were shaken for 2 min. On days when OD was not measured, plates were shaken for 2 min, as well. The experiment flow slightly differed for heat shock treatment. In essence, the first OD reading was taken on day 0 after L1 larvae were dispensed onto the experimental plates, and heat shock treatment (30 min at 37 °C) was administered at the same larval stage as MMS and H_2_O_2_ stressors, but the additional liquid was not added to the experimental plates. The last OD reading was taken on the same day for every treatment. Amount of bacteria eaten was used as a fitness proxy following Elvin and colleagues [28].

Each double mutant was measured in at least 8 replicates (2 biological replicates (runs) on two different dates with 4 technical replicates (4 wells) in each biological replicate). In each run, single-mutant a (mutant worm on HT115 (DE3) control bacteria with empty vector), single-mutant b (wild type worm on RNAi bacteria), and control animals (wild-type N2 strain on HT115) were measured in at least four replicates.

### 2.6. Assay Quality Control, Data Standardization, and Data Quality Control

Only wells with the first OD reading between 0.745 and 0.905, animal density between 10 and 30 individuals/per well, and no contamination were used in further analysis. Bacterial density above 0.905 caused worm mortality, whereas density lower than 0.745 was too low to sustain worms for the whole duration of experiment.

The final OD measurement was standardized by the first day OD reading of that well, as the first reading could span from 0.745 to 0.905 and then extracted from the first day reading (now equal to 1), to calculate how much bacteria were consumed, which was our fitness proxy. Each mutant fitness was then standardized by wild-type fitness from the same run to correct for batch effect. We call this standardized fitness proxy fitness throughout the text.

The coefficient of variation (CV) for the fitness of each mutant from all the readings for that mutant (separately for each condition) was calculated, and if the CV value was higher than 20%, we excluded the record with the highest deviation from the mean value and calculated CV again. The procedure was repeated until the CV was smaller than 20% or only one record was left. In that last case, mutants were dropped from further analyses.

### 2.7. S-Score Calculation

S-score (a measure of epistasis) for all double mutants, for each well, was calculated as a strictly standardized mean difference [29,30]:

S-score = (Vobs − Vexp)/VAR(1)

where Vobs is the observed fitness of double mutant, Vexp is the expected fitness of double mutant, calculated as a product Wa × Wb, where Wa is the fitness of single-mutant a (mutant strain on control bacteria) and Wb is the fitness of single-mutant b (wild-type strain on RNAi bacteria), and variance VAR = sqrt (VARab + VARa × Wb^2^ + VARb × Wa^2^).

Next, mean S-scores for each double mutant were calculated.

### 2.8. S-Score Significance

We considered two methods of assessing the significance of epistatic interaction. The first was based on *p*-value (FDR corrected)—if a particular S-score differed significantly from zero, we called it epistatic. The second method assumed that S-scores below −1.28 and above 1.28 are significant, which was one of the thresholds proposed by Zhang [31] to present moderate genes effects. We decided to choose the second approach since it was more conservative. For control data from heat shock experimental block, they showed less significant interactions (45) than the *t*-test (for corrected *p*-value < 0.05—223 interactions), but it matched the amount found in another high-throughput screening—4%. What is more, almost all interactions discovered by the 1.28 threshold were also considered significant by the first method, i.e., 42 out of 45.

### 2.9. S-Score Distribution Comparison

To check if epistatic interactions differ between standard and stress conditions, we used a paired *t*-test to check if there is a significant difference in double-mutant S-scores. Additionally, we used the randomization method. We counted overlapping areas between S-score distributions in standard and heat shock, using the R package ‘overlapping’ [32]. Next, we combined S-scores from both conditions (assuming that all S-scores come from the same distribution) and sampled (with return) two samples of n values (n is equal to the number of epistatic interactions checked in relevant conditions) and counted overlapping areas of obtained distributions. We repeated this procedure 1000 times to obtain the distribution of overlapping areas. Finally, we compared our observed overlap with the obtained distribution.

### 2.10. Differential Epistasis Calculation

We calculated differential epistatic score (D-scores) as the difference between S-score in each stress condition and in corresponding standard conditions; D-score = S-score_stress condition_ − S-score_standard condition._ We used the method described by S. Bandyopadhyay et al. [9], with modifications to check for D-score significance. First, we assessed how repeatable are measurements of S-scores in standard conditions. For that, we checked the differences between S-scores for the same double mutants from different runs. Obtained distribution was used as null distribution. Next, we compared null distribution with differences between double mutants S-scores measured in standard and stress conditions. Each difference that was in 2.5% of the lowest and highest values of null distribution was considered statistically significant.

### 2.11. Sign Epistasis and Reciprocal Sign Epistasis

We analyzed all gene–gene interactions that were assumed to be a true epistatic interaction (higher than 1.28 or lower than −1.28) and were repeated at least three times for the presence of sign and reciprocal sign epistasis. Data from each condition were analyzed separately. In the first step, we checked if the fitness of any of the two single mutants differed significantly from the wild type (fitness = 1) using a *t*-test. Next, Wilcoxon tests were used to check if the fitness of the double mutant is significantly different from any of its corresponding single mutants. *p*-values from all tests were corrected for multiple comparisons, using the FDR method.

Gene–gene interaction was considered as sign epistasis if one of the following criteria were met: (i) the fitness of both of the single mutants is significantly lower than 1, and the fitness of the double mutant is significantly higher than that of at least one of the single mutants; (ii) the fitness of at least one single mutant is significantly lower than 1, and fitness of double mutant is significantly higher than the fitness of that single mutant; (iii) the fitness of both of the single mutants is significantly higher than 1 and the fitness of the double mutant is significantly lower than at least one of single mutants; or (iv) the fitness of at least one single mutant is significantly higher than 1, and fitness of double mutant is significantly lower than the fitness of that single mutant.

Additionally, gene–gene interaction was considered as reciprocal sign epistasis if one of the criteria was met: (i) the fitness of both single mutants is significantly lower than 1 and the fitness of double mutant is significantly higher than the fitness of both single mutants, or (ii) the fitness of both single mutants is significantly higher than 1 and the fitness of double mutant is significantly lower than the fitness of both single mutants.

### 2.12. Data Availability

The authors affirm that all data necessary for confirming the conclusions of the article are present within the article, figures, and tables.

## 3. Results

We screened over 5000 pairwise interactions in *C. elegans* by feeding single-mutant worms with RNAi bacteria (Supplementary Table S3). We tested these interactions in three stressful conditions—heat, oxidative (H_2_O_2_), and mutagenic (methyl-methanesulfonate, MMS) stress. Our experimental design consisted of two separate blocks, one involving heat shock (1195 pairwise interactions tested) and standard laboratory conditions (Standard-H; 1195 pairwise interactions tested), and the other one H_2_O_2_ (1134 pairwise interactions tested), MMS (1144 pairwise interactions tested), and standard treatment (Standard-OM; 1148 pairwise interactions tested).

As a measure of epistasis, we calculated the S-score (strictly standardized means difference between observed and expected fitness of double mutant, see Materials and Methods. We considered interaction significant if its S-score value was higher than 1.28 (positive epistasis) or lower than −1.28 (negative epistasis).

**Figure 1:**
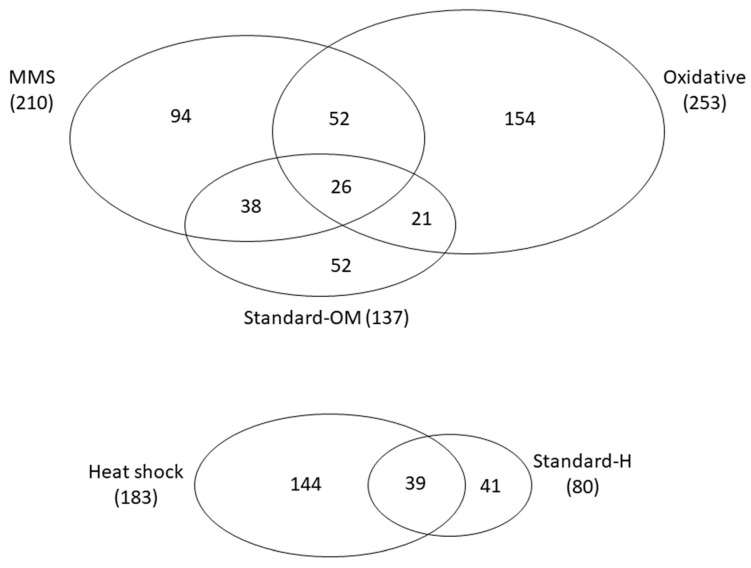
The number of interactions shared between treatments with respect to experimental blocks.

We detected 7% and 12% of significant epistatic interactions in Standard-H and Standard-OM conditions, respectively. In harsh conditions, the number of epistatic interactions increased; in the heat shock treatment, 15% of tested gene pairs exhibited epistatic interactions: MMS, 18%; H_2_O_2_, 22% (Figure 1).

### 3.1. Direction of Epistasis

Out of all significant epistatic interactions in Standard-H conditions, 45 were significantly positive and 35 negative, whereas in Standard-OM conditions, we found 55 positive and 82 negative interactions. In MMS treatment, the same amount of interactions had positive and negative signs (105). In two harsh conditions, there were more positive than negative interactions. In H_2_O_2_ treatment, we observed 134 positive and 119 negative interactions, and in heat shock treatment, we observed 151 positive and 32 negative interactions.

Overall, the sign of epistasis was slightly negative in two conditions: in Standard-OM condition with a mean S-score value of −0.06, and in MMS treatment with a mean S-score of −0.04, but only in Standard-OM conditions, this value was significantly lower than zero according to *t*-test (*p*-value 0.005 vs. 0.267 in MMS treatment). In three conditions, the overall sign of epistasis was significantly higher than zero: Standard-H (mean 0.09, *p*-value < 0.001), heat stress (mean 0.37, *p*-value < 0.001), and H_2_O_2_ treatment (mean 0.10, *p*-value 0.003).

### 3.2. Differential Epistasis

To see how the environment changes genetic interactions, we calculated the differential epistasis score (D-score), which is a difference between S-scores from treated and untreated groups. We observed an overall positive differential epistatic effect in all tested environments. The mean D-score in heat shock was 0.30, with 0.16 in oxidative stress, and 0.03 in MMS. Despite having the highest mean D-score, the lowest number of significant D-scores (higher than 1.28 or lower than −1.28) was found in heat shock (63 positive and 33 negative). In oxidative stress, we discovered the highest number of significant differential epistatic interactions, with 178 positive and 158 negative, and in MMS, there were 115 positive and 101 negative D-scores (Supplementary Table S4).

### 3.3. Persistence of Epistasis across Environments

**Figure 2:**
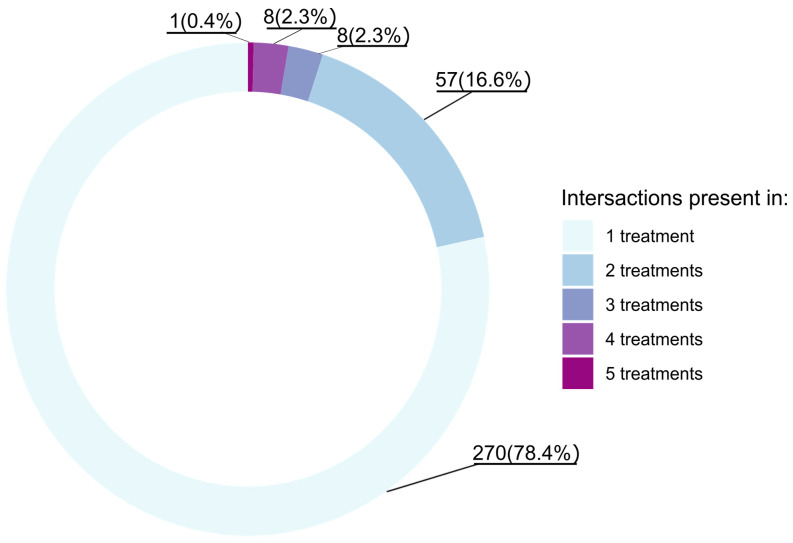
Percentage of interactions shared between treatments. As results come from two experimental blocks, S-scores < −1.96 and >1.96 are considered significant.

We discovered that among all significant interactions (with S-scores higher than 1.96), only one was shared between all conditions, whereas 78% of interactions were condition-specific (Figure 2).

**Figure 3:**
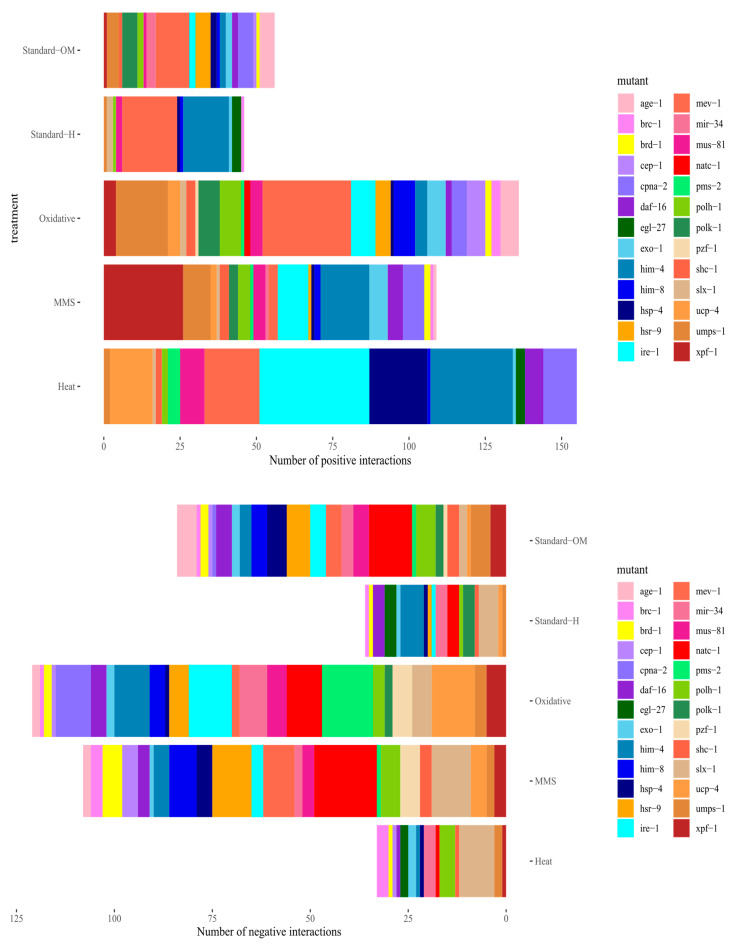
Barplot showing proportion of particular genes which contribute to significant epistatic interactions.

Within 78 interactions shared between MMS and Oxidative stressors, 30 exhibited a difference in the sign of epistasis; 16 of the interactions were negative in MMS and became positive in oxidative stress, whereas 14 were the other way around. Standard-OM shared only eight cases of interactions that changed the sign of epistasis with Oxidative and six with MMS. Only one interaction (*mir-34: egl-27*) was consistently negative in stressors, in contrast to standard conditions. In heat shock and Standard-H, there were only two such cases; both changed the sign to positive in heat stress (Supplementary Table S5). As shown in Figure 3, in most conditions, there were hub genes enriched in the number of interactions, but they changed depending on the environment.

### 3.4. Sign and Reciprocal Sign Epistasis

**Figure 4:**
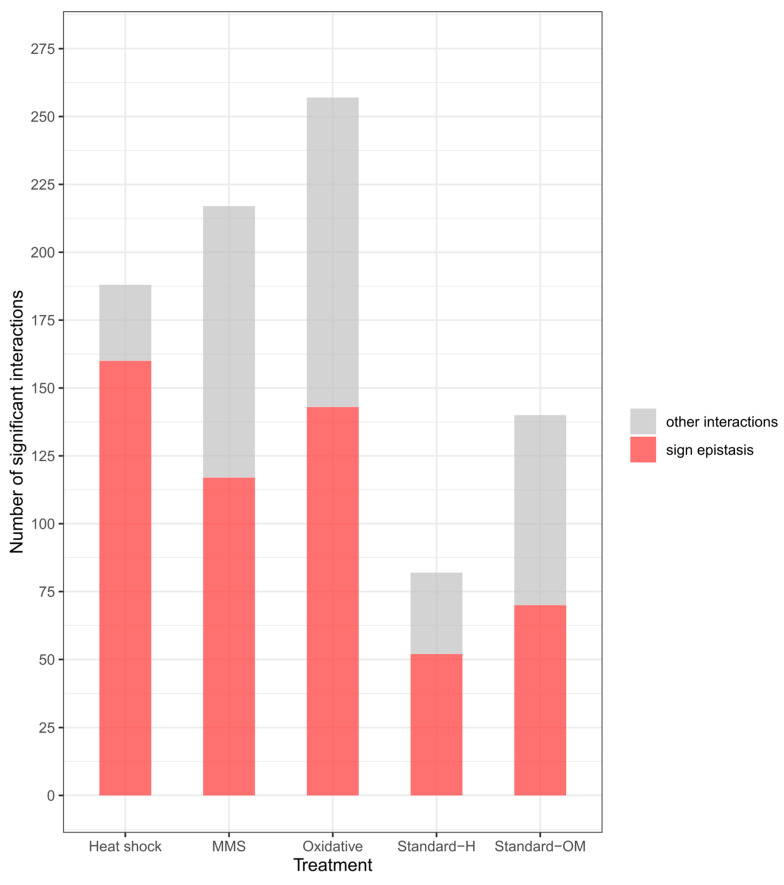
Proportions of sign epistasis interactions to the rest of the significant interactions (S-score < −1.28 and >1.28) were found in each treatment. Sign epistasis—either of the mutants’ fitness effects is opposite to the double mutant.

Sign and reciprocal sign epistasis are unique examples of genetic interactions. Sign epistasis can be considered a special case of positive or negative epistasis in which the effect of single mutants has opposite effects when combined with additional mutation. Reciprocal sign epistasis is a rather rare event in which the fitness of a double mutant is increased/decreased, despite both single mutations having the opposite effect on fitness. We found 63 examples of sign epistasis in Standard-OM and 44 in Standard-H conditions. In treatment groups, the highest number of such interactions was found in heat shock (144), then in oxidative stress (116), and only 80 were found in MMS treatment. What is interesting is that 144 sign epistasis interactions in heat shock account for almost 80% of all significant interactions in this treatment, whereas in other conditions, sign epistasis oscillates around 50% of all interactions (Figure 4).

We did not find any cases of reciprocal sign epistasis in standard conditions, MMS, and heat shock treatment. However, we observed three reciprocal sign interactions in H_2_O_2_. In all of those cases, the effect of double mutations is beneficial despite the deleterious effect of both single mutations.

## 4. Discussion

**Figure 5:**
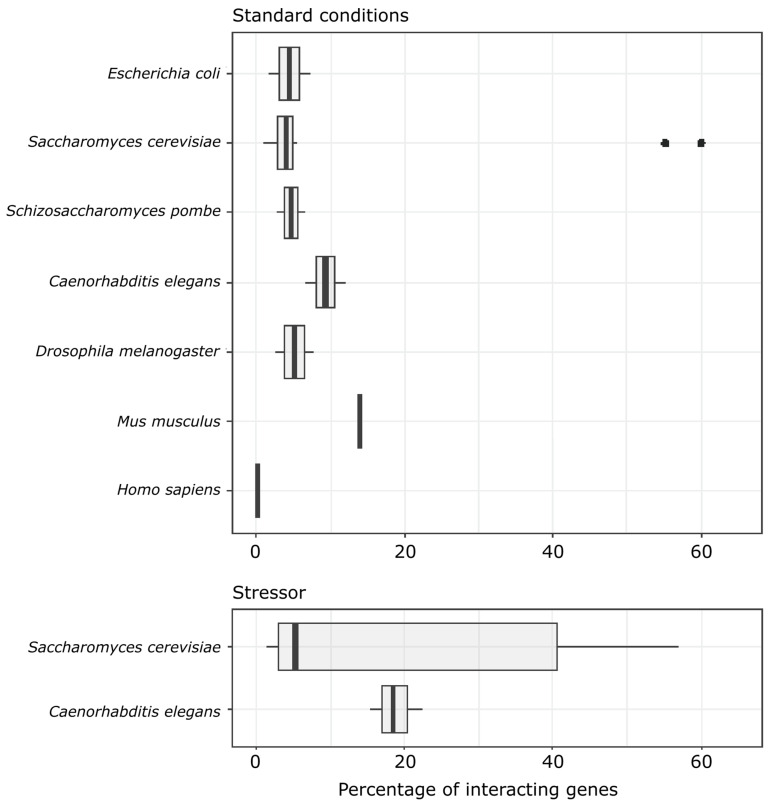
Amount of epistatic interactions found in other quantitative epistatic studies (bold, vertical line represents median value, left and right hinges correspond to first and third quartile, whiskers extend from hinges no further than 1.5 interquartile range of the hinges, dots are outliers). Detailed data are presented in Supplementary Table S6.

There is an ongoing struggle to discover whether there is a universal pattern of epistasis for all living organisms. So far, most experimental studies show that epistasis covers from 0.14 to 14% of tested gene–gene interactions (Figure 5). Limited data about interactions in stressful conditions point that only in some cases does the amount of epistasis increase, and we cannot really see any consistent effect (Figure 5). In 2006, Sanjuan and Elena [33] tried to correlate the sign of epistasis with genomic complexity, showing that the more complex an organism, the more prevalent the enhancing epistasis. Our results disagree with that statement, since the mean epistatic effect in both standard conditions did not differ from zero in our study. However, what needs to be considered is that our knowledge about genetic interactions in higher organisms is rather scarce. The abovementioned study by Sanjuan and Elena [33] based its main conclusions on only five species (one virus, one prokaryote, and three eukaryotes, and the latter ones included two species of fungus and one animal), so the differences between those studies and our is not surprising, and they show a clear need for further studies related to the topic before robust conclusions can be drawn. High-throughput studies on epistasis are vastly monopolized by unicellular organisms or cell cultures (Supplementary Table S6). Although there is a growing body of studies showing that most complex traits are specified by multiple loci and epistatic interactions between those loci are common [34,35], the specific questions on the mean sign of epistasis and how it changes with environmental conditions are still poorly understood. Our research provides valuable insight into the genetic dependencies of multicellular organisms with quantitative results.

When it comes to both the amount and predominant sign of epistasis, oxidative and heat shock treatment follow the same pattern, whereas the mutagenic stressor (MMS) seems to resemble a more standard conditions trend. In oxidative stress and heat shock treatment, we found more interactions than in standard and MMS conditions. Our results seem to agree with the results of recent studies, which also find that some stressors can increase the number of genetic interactions. Two studies on *Saccharomyces cerevisiae* found a similar pattern. One observed more interactions in treatment which affected respiratory growth (but not in other stressors, including heat shock, [36]), whereas the other found slightly more interactions in zymolyase and H_2_O_2_ but not in sorbitol [37]. This implies that interactions are condition-dependent, and not uniform across stressors.

But the main question that appears is this: why does a stressful environment induce more interactions to show up? It is known that the environment does not only act on phenotypic plasticity but can also alter genome activity. Many external factors can up- or down-regulate genes’ expression [38]. For example, heat-shock protein (hsp) genes are well known to be affected by a variety of stressors, which results in different expression patterns [39]. Therefore, we could argue that exposure to a stressor is increasing the number of interacting loci in our experimental setup. If so, we observe a gene–gene interaction in standard conditions, but rather a higher-order interaction in a harsh environment.

Studies on yeast show that while only 20% of genes are essential in standard conditions, 97% of genes are essential in at least one tested environment [40]. Every stressor appears to have a different effect on the genomic pathways which manifests in a variety of epistatic interactions exhibited in our experiment. Even though our results call attention to the similarities between heat and oxidative stress, only 8.5% of interactions are shared among them. We have found that only one interaction was shared among all of the tested treatments (*hsp-4 x mus-81*), while over three quarters of interactions are unique for a particular condition (Figure 2). Jaffe et al. [36] found similar results in their studies; with every added stressor, the number of interactions increased. What is interesting is that the only universal interaction found in our study (*hsp-4 x mus-81*) was positive across all environments. Both of those genes are associated with GO terms, i.e., response to stimulus and hydrolase activity, and both influence growth.

Our study detected that in oxidative stress and heat shock treatment, the majority of interactions were positive, with the mean value of the S score being ten-fold higher than in MMS and standard conditions. The studies conducted so far report contradictory results of such analysis; however, they vary in terms of how vast the datasets were and what organisms were used. A study which covered a large number of genes and tested epistasis in different treatments showed that positive interactions are prevalent [8]. However, they did not detect significant differences between stressors and standard conditions. The flux balance analysis study also provided information about interactions being more positive in harsh (here: food-limited) conditions [15].

**Figure 6:**
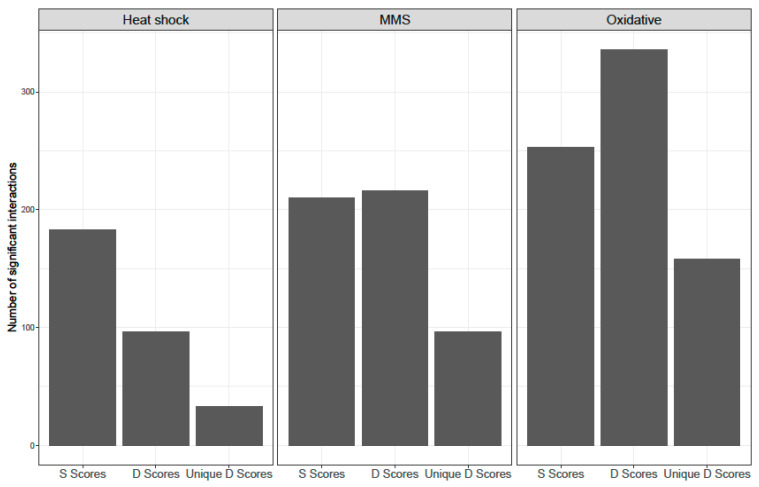
Results of differential epistasis analysis for each treatment. D-scores are calculated as the difference between control and stress conditions. Unique D-scores—interactions uncovered only in D-score analysis but not observed as significant S-scores.

An alternative way to present change in epistatic effect from standard to stressful conditions is by calculating the D-score (differential epistatic score). All mean D-scores were positive (0.03–0.3), which indicates that interactions tend to be more positive in harsh conditions. Regardless, the heat shock mean D-score was slightly lower than the mean S-score value (0.3 vs. 0.39) and the overall number of significant S-score interactions was almost two times higher. In MMS, we detected slightly more positive D-score than S-score interactions, while in H_2_O_2_ treatment, the number of both positive and negative D-scores exceeds the number S-scores. Also, D-scores enabled us to uncover interactions that were not spotted/not considered significant, compared to when we only calculated S-scores. Out of all interactions that were not significant, according to the S-score value, 3% had significant D-scores in heat shock and 10% in MMS. Oxidative stress stands out with almost 18% unique D-score interactions (Figure 6).

Several evolutionary aspects are affected or dependent on epistasis. However, the discussion of whether epistasis should be considered an important force in the context of long-term evolution is still unresolved. Multiple studies examined this subject but a consensus has not been reached. Few papers argue that genetic interactions have little effect on evolutionary dynamics and could be neglected in terms of the long-term response to selection [41,42,43]. Yet others point out that under specific circumstances, such as directional epistasis, genetic interactions could contribute significantly to evolution/additive genetic variance [3,44,45]. Similarly, recent studies shed light on predicting postzygotic isolation events using information about the directional selection. The models show that positive interactions within the population can contribute to heterosis. But what is more important is the effect that those interactions have on divergence. It appears that at the beginning break of interacting alleles has a small effect on hybrids’ fitness, but it amplifies in time. This continues up to a point, where the disruption effect can generate a fitness loss of an order of magnitude higher than that which new, negative interactions would cause. Between-population interactions were found to have more negative effects and act more randomly. However, the authors do point out that the strength of selection and drift are important factors that strongly influence evolutionary fate [46]. On the other hand, a study performed on Solanum hybrids found prevalent positive interactions in introgression lines, which could lead to slowing down the isolation process [47]. Normally, Dobzansky–Muller incompatibilities provide a great explanation of how reciprocal sign interactions can cause hybrid sterility [48]. Even though reciprocal sign epistasis is well acknowledged in fitness landscape studies [49], we are unable to state whether this type of genetic interaction is a rare event or not, since only a few studies tested it [50,51,52,53]. In *E. coli*, it was found that 40% of mutations were sign epistasis, but only one was reciprocal sign epistasis (0.02%) [50], whereas in mammals, reciprocal sign epistasis might comprise even 2.9% [53]. This would suggest that in more complex organisms, reciprocal sign epistasis is more frequent. While we found only three such cases in our study, it actually comprises 0.35% of all found interactions. Although *C. elegans* is a multicellular organism, it is much less complex than mammals, so those data would fit on the axis of the relationship between complexity and reciprocal sign epistasis occurrence. But our results might also be specific to our dataset.

In general, the question of how much epistasis can influence evolution through selection is still unanswered, as our knowledge of the number and prevalent sign of epistasis remains limited.

## Data Availability

Data are contained within the article and Supplementary Materials.

## Supplementary Materials

The following supporting information can be downloaded at: https://www.mdpi.com/article/10.3390/genes14112080/s1, Table S1. List of strains used in the experiment and their sources. Table S2. List of genes targeted via RNA interference method. Table S3. List of all tested interactions with counted S-scores. Table S4. Differential epistasis analysis results. Table S5. Comparison of all significant interactions shared between treatments (within tested experimental block). Table S6. Sourced literature data for quantitative epistasis [9,10,16,17,18,19,20,21,36,37,54,55,56,57,58,59,60,61,62].
